# Phenotypic and genotypic variations among three allopatric populations of *Lutzomyia umbratilis*, main vector of *Leishmania guyanensis*

**DOI:** 10.1186/s13071-015-1051-7

**Published:** 2015-09-04

**Authors:** Moises Thiago de Souza Freitas, Claudia Maria Ríos-Velasquez, César Raimundo Lima Costa, Carlos Alberto Santiago Figueirêdo, Nádia Consuelo Aragão, Lidiane Gomes da Silva, Marcus Vinicius de Aragão Batista, Teresa Cristina Leal Balbino, Felipe Arley Costa Pessoa, Valdir de Queiroz Balbino

**Affiliations:** Departament of Genetic, Federal University of Pernambuco, Avenida Professor Moraes Rego S/N,Cidade Universitária, Recife, Pernambuco 50732-970 Brazil; Departament of Microbiology, Research Center Aggeu Magalhaes, Avenida Professor Moraes Rego S/N,Cidade Universitária, Recife, Pernambuco 50732-970 Brazil; Laboratory of Infectious Disease Ecology in the Amazon, Instituto Leônidas e Maria Deane - Fiocruz Amazônia, Rua Terezina, 476, Adrianópolis, Manaus, Amazonas 69.057-070 Brazil; Department of Biology, Federal University of Sergipe, Av. Marechal Rondon s/n - Rosa Elze, São Cristóvão, Aracajú, Sergipe 49100-000 Brazil

**Keywords:** *Lutzomyia umbratilis*, Species complex, Phylogenetic analysis, Geometric morphometry

## Abstract

**Background:**

In South America, *Lutzomyia umbratilis* is the main vector of *Leishmania guyanensis*, one of the species involved in the transmission of American tegumentary leishmaniasis. In Brazil, *L. umbratilis* has been recorded in the Amazon region, and in the state of Pernambuco, Northeastern region, where an isolated population has been identified. This study assessed the phylogeographic structure and size and shape differences of the wing of three Brazilian populations.

**Methods:**

Samples of *L. umbratilis* were collected from Rio Preto da Eva (north of the Amazon River, Amazonas), from Manacapuru (south of the Amazon River), and from the isolated population in Recife, Pernambuco state. These samples were processed to obtain sequences of the Cytochrome Oxidase I mitochondrial gene. Geometrics morphometry analysis of the right wing shape of the three populations was made using discriminate canonical analysis.

**Results:**

Phylogenetic analysis revealed the presence of two distinct monophyletic clades: one clade comprised of the Recife and Rio Preto da Eva samples, and the other clade comprised of the Manacapuru samples. Comparing the Manacapuru population with the Recife and Rio Preto da Eva populations generated high indices of interpopulational divergence. Geometric morphometry analysis indicated two distinct groups between the studied populations. Canonical variate analysis of wing shape indicated that Rio Preto da Eva population is significantly closer to Recife population, and both populations were genetically distant from Manacapuru.

**Conclusion:**

The polymorphic sites and geometric morphometry analysis indicate that the distance, lack of continuity and environmental differences have not modified the ancestral relationship between Recife and Rio Preto da Eva populations. The genetic and morphological similarities shared by the Recife and Rio Preto da Eva populations suggest that these populations are more closely related evolutionarily. These results confirm the existence of an *L. umbratilis* species complex in the North and Northeast regions.

## Background

*Lutzomyia umbratilis* (Diptera: Psychodidae) is the main vector of *Leishmania guyanensis,* one of the pathogenic agents of American tegumentary leishmaniasis (ATL) [1–3]. This species is found in Brazil, Bolivia, Colombia, French Guyana, Guyana, Peru, Suriname and Venezuela [4, 5]. In Brazil, *L. umbratilis* is widely distributed in the Amazon basin and there is an isolated population in remnants of the Brazilian Atlantic rain forest in the state of Pernambuco, Northeastern Brazil [4–6]. Of the states in the Northeastern Region, Pernambuco has the third highest number of ATL cases reported in Metropolitan Regions [7]. On the other side of Brazil, in the Amazon region, the presence of *L. umbratilis* naturally infected with *Leishmania guyanensis* has been recorded on the east side of the Negro River and the north side of the Amazon River, but not on the south side [8]. In the state of Amazonas, 12,005 cases of ATL were registered between 1994 and 2005; in 2003, 60.18 % of ATL cases occurred in the city of Manaus, where *L. umbratilis* is recognized as the most significant vector [9, 10].

Considerable geographic distance separates the *L. umbratilis* populations of the North and Northeast Regions. The low dispersal capacity and geographic isolation of sand flies may result in population structuring, which is amplified by the presence of abiotic barriers [11–13]. The *L. umbratilis* populations of the North and Northeast Regions are separated by distance and abiotic barriers significant enough to suggest that these individuals may have evolved into genetically differentiated populations.

Bionomical (*e.g.* fecundity, fertility, and duration of larval development) and genetic differences between *L. umbratilis* populations on the north and south sides of the Amazon River, suggest that these populations might represent a species complex comprised of at least two incipient species [14, 15]. In light of this possibility, this study aimed to assess the evolutionary relationships between populations of *L. umbratilis* from the states of Pernambuco and Amazonas.

## Methods

### Field collection and identification of phlebotomine sand flies

Field collections were done in Rio Preto da Eva (north of the Amazon River, Amazonas) (2°50’50”S/59°56’28”W), from Manacapuru (south of the Amazon River) (3°12’41”S/60°26’20) municipalities, located in the Amazonas State, North Region of Brazil, and in the Atlantic Forest Ecological Reserve of Dois Irmãos (8°03’14”S/34°52’52”W) in Recife Municipality, Pernambuco State, Northeast Region of Brazil.

Adult specimens were collected by suction in tree trunks using CDC light traps. The samples were conserved in 95 % alcohol, at -20 °C. The genitalia of the individuals were slide mounted and stored as a voucher, and the sand flies were identified using the keys of Young and Duncan [4].

### DNA extraction, PCR, and sequencing

In all, 201 specimens of *L. umbratilis* were used in the experiments geometric morphometry and molecular biology. For molecular analysis, 129 specimens of *L. umbratilis* were used: 36 from Manacapuru (24 males and 12 females); 38 from Rio Preto da Eva (15 males and 23 females); and 55 from Recife (25 males and 30 females).

Genomic DNA extraction was carried out using Chelex®100 (BioRad, Berkeley, California, USA), according to Lima Costa-Junior *et al.* [16]. A fragment of 597 bp of the Cytochrome Oxidase I (COI) was amplified by PCR using the universal primers CI-J-1632 (+): 5-TGATCAAATTTATAAT-3 and CI-N-2191 (−): 5-GGTAAAATTAAAATATAAACTTC-3, described by Simon *et al.* [17]. Amplification reactions were done using the Mix Go Taq Colorless kit, according to manufacturer specifications (Promega® Fitchburg, Wisconsin, USA). PCR products were visualized in 1 % agarose gel through UV light and they were purified using the Wizard® SV Gel and PCR Clean-Up System kit (Promega® Fitchburg,Wisconsin, USA). Sequencing was carried out in ABI 3500 automatic sequencer (Applied Biosystems, Cleveland, Ohio, USA). Only sequences with a Phred score above 30 were used in the analysis. Contig assembly was carried out using CodonCode Aligner (CodonCode Corporation). Local alignments were done using BLAST [18]. All new sequences produced in this study have been deposited in GenBank under accession numbers: KM407009 to KM407137.

### Phylogenetic analysis

Nucleotide sequences were aligned using Muscle [19], incorporated in MEGA v. 5.0 [20]. Phylogenetic analysis was carried out with the Maximum Likelihood criterion using PhyML [21]. The evolutionary model that best fits the data was HKY + G, according to jModelTest [22]. The consistency of the branches was assessed using 1000 bootstrap replicates. *Lutzomyia longipalpis, Lutzomyia migonei* and *Phlebotomus papatasi* were used as the outgroup.

### Divergence time estimate

The time at which the two main clades diverged (into the Manacapuru and Rio Preto/Recife isolates) was estimated with a relaxed molecular clock using BEAST v. 1.7.5 [23]. The analysis was done for 10,000,000 generations, sampling every 1000 generations. The nucleotide substitution model used was HKY + G. An uncorrelated lognormal clock and a tree prior with *Yulebirth* process were used. The tree with the higher clade Bayesian Posterior Probabilities (BPP) was obtained with TreeAnnotator v. 1.7.5 (available in BEAST package), using the *meanhights* option and 10,000 trees as burnin to assure stationarity.

### Genetic diversity

Intra-population genetic diversity was measured by the haplotype and nucleotide diversity, K value (number of genetic groups), number of polymorphic sites, and number of transitions and transversions using DnaSP v. 4.0 [24] and Arlequin v. 3.5 [25]. The frequencies of polymorphic sites were also assessed using WebLogo tool (http://weblogo.berkeley.edu/logo.cgi).

Tajima’s D neutrality test, Fu’s *Fs* statistics, and the Mismatch distribution were performed using Arlequin v. 3.5 [25]. The Raggedness index (r) and the Sum of squared deviations (*SSD*) were used in order to validate the model of recent expansion obtained using the Mismatch distribution. Genetic differentiation was assessed with the pairwise fixation index *F*_st_ using Arlequin v. 3.5 [25].

The average number of substitutions per site among populations (*D*_xy_), the total number of substitutions per site among populations (*D*_a_), the number of shared polymorphisms among populations (*S*_s_), and the number of fixed differences among populations (*S*_f_) were calculated using DnaSP v. 4.0 [24].

The haplotype network was created with NETWORK v. 4.6 (www.fluxus-engineering.com) using the Median-joining method [26] to verify the level of haplotype sharing and distribution frequency among the populations.

### Population structure

Genetic structure analysis was performed using Structure v. 2.3 [27]. Interactions were carried out with 20,000 interactions of burning, followed by 200,000 generations of Markov Chain Monte Carlo, adjusted 1 to 10 for each “K” population. Ad hoc quantity ∆K [28] was used to determine the most accurate number of “K” groups.

### Geometric morphometry of the wings

The right wings of adult females of *L. umbratilis* from the municipalities of Rio Preto da Eva (*n* = 18), Manacapuru (*n* = 27) and Recife (*n* = 27) were dissected with forceps and mounted between microscope slide and cover slide using Berlese fluid. Images of each wing were obtained at 4× magnification using a JVC KY-F55 digital camera coupled to a Leica DM 1000 optical microscope.

**Fig. 1:**
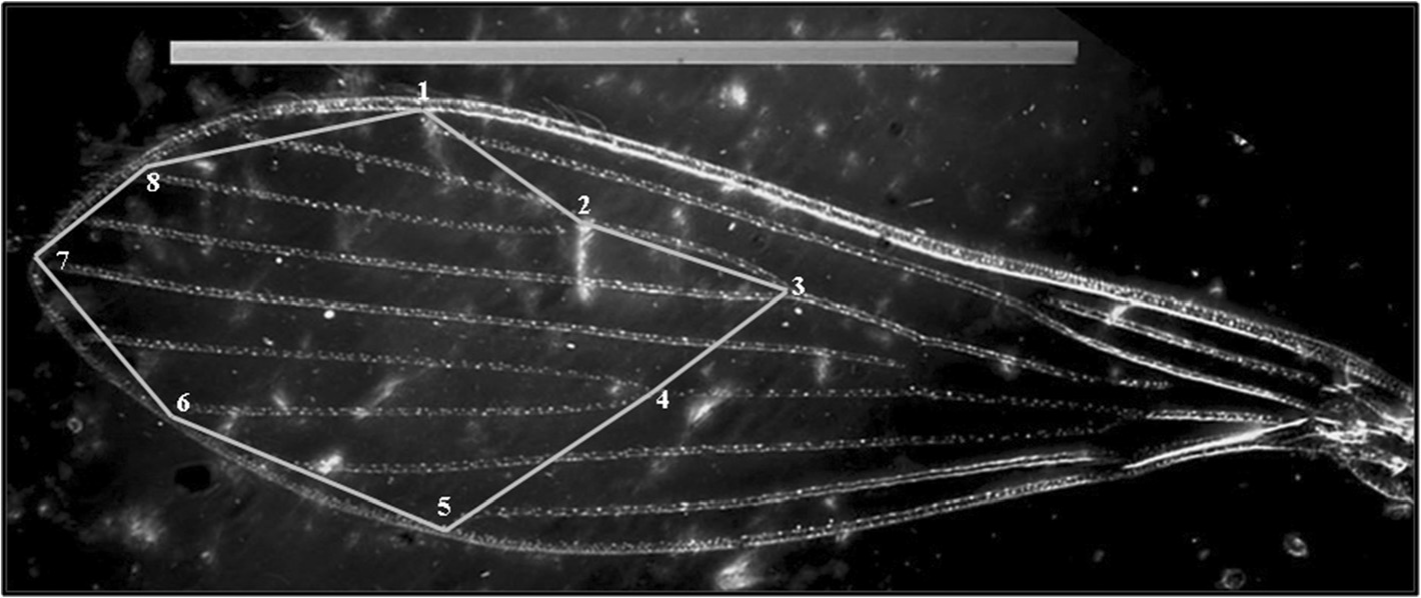
Photograph of the right wing of *L. umbratilis* female, showing the position of landmarks (LM). The numbers indicate the order of LM collection; their junction forms a geometric shape. Scale: 1 mm

A total of eight ‘type I’ landmarks (LM) were identified (Fig. 1), all in the wing venation intersections [29]. The coordinates of wing landmarks were digitized using the TpsDig2 v 2.18 (QSC - James Rohlf). The shape variables were calculated with *tpsRelW* v. 1.46. MOGwin v. 0.82 was used to analyze the shape variables by the generalized Procrustes analysis superimposition algorithm (GPA) [30].

The GPA algorithm was used to extract the centroid size; allowing size and shape to be analyzed separately. Size variations were analyzed using the Kruskal-Wallis test. The effect of allometry was analyzed by regression analysis using the coordinates of the landmarks and the centroid size for the three populations. Discriminant analysis of the canonical variables was performed to compare the shape with the overall mean wing size of each population, and the Mahalanobis distances were calculated using 10.000 permutations. To the morphometric statistical analyses we used the softwares TpsUtil 1.60, TpsDig2 v 2.18 (QSC - James Rohlf), TpsRelw 1.54 (QSC - James Rohlf), Tet, MorphoJ 1.06 and Past Program (Paleontological Statistics) v. 2.00.

## Results

### Molecular analysis

**Fig. 2:**
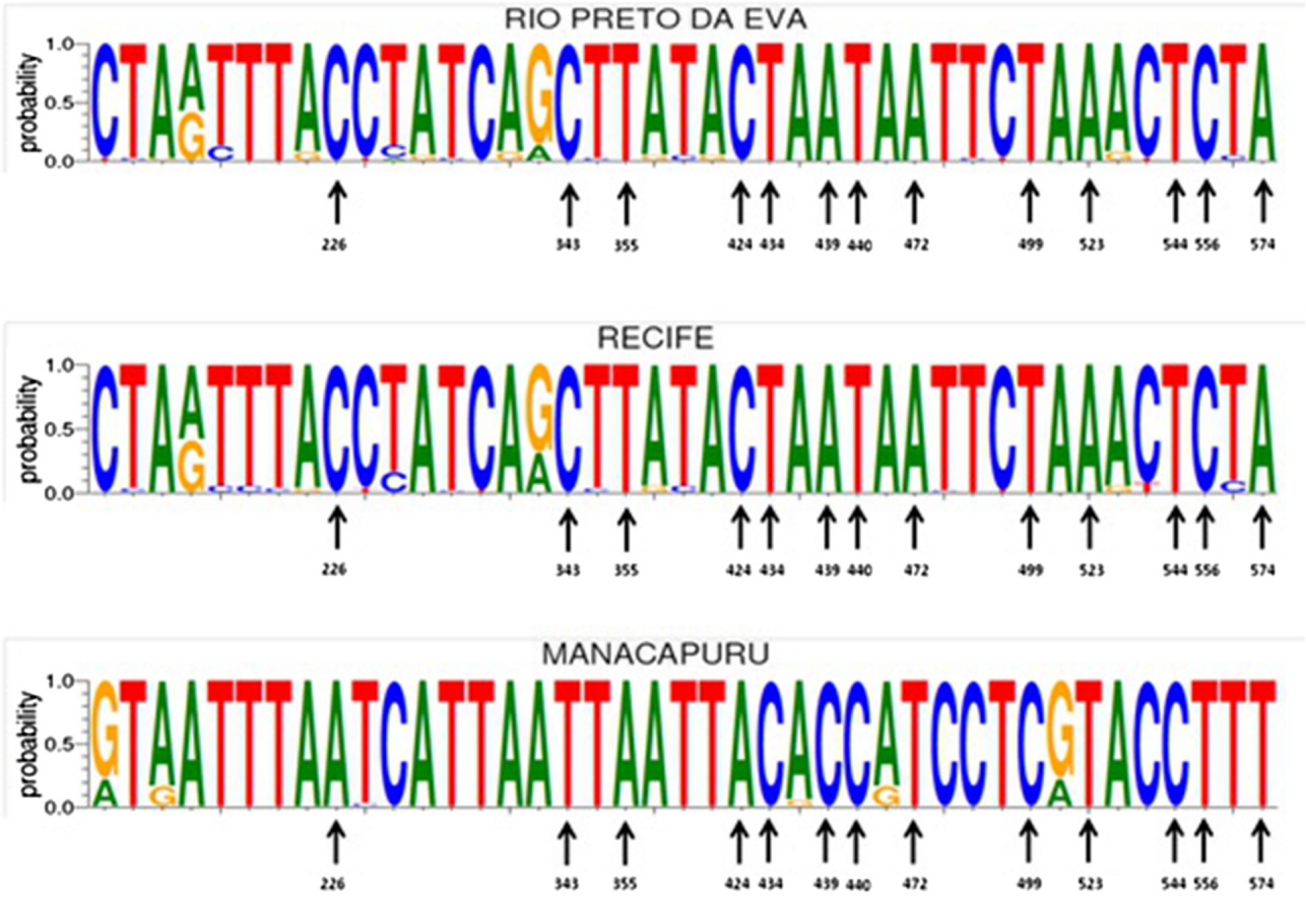
Schematic representation of polymorphisms of a fragment of 597 bp of the gene Cytochrome Oxidase I using Weblog. Shown are the sequences obtained from *L. umbratilis* collected in Recife, State of Pernambuco, Rio Preto da Eva and Manacapuru, State of Amazonas, Brazil. Font size is indicative of the frequency of a nucleotide at any given site. Fixed (black arrows)

Altogether, 129 specimens of *L. umbratilis* were analyzed. Across the region of sequence analyzed (597 bp), 66 (12.2 %) polymorphic sites observed; these were comprised of 42 (~63.6 %) parsimony-informative sites and 24 (~36.4 %) singletons. Among the polymorphic sites, 84.9 % of the nucleotide substitutions were transitions and 15.1 % were transversions. Analysis of the polymorphic sites identified 13 fixed single nucleotide polymorphisms (SNP) within the 597 bp fragment of COI used in our analyses (Fig. 2). Indels and non-synonymous nucleotide substitutions were not found.

**Fig. 3:**
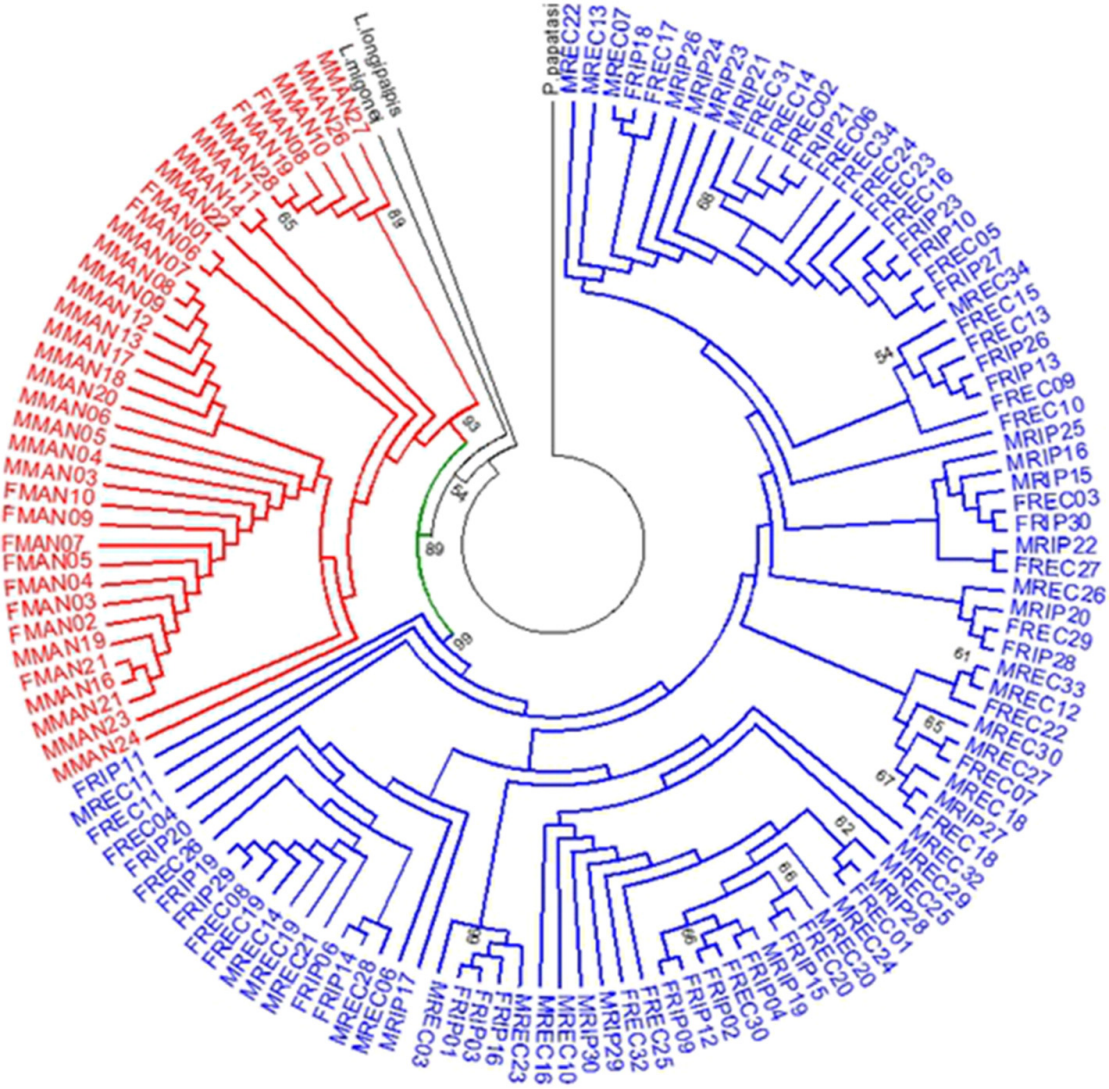
Maximum Likelihood Tree obtained with the GTR + I model, showing results using 597 bp from *L. umbratilis* period markers. The sequences corresponding to the gene period in *Phlebotomus papatasi* (JN172078), *Lutzomyia longipalpis* (GU909503) and *Lutzomyia migonei* (GU909508) were used as external groups. The localities are: Manacapuru (M/FMAN) and Rio Preta da Eva (M/FRIP) in the State of Amazonas, and Recife (M/FREC) in the State of Pernambuco, Brazil. Note that this topology was able to consistently separate the two monophyletics clades: Clade I and Clade II, with 93 and 99 % bootstrap values, respectively

The Maximum Likelihood analysis indicated difference, revealing two distinct clades, well-supported with bootstrap values of 93 and 99 %, respectively (Fig. 3). This result indicates that the Recife and Rio Preto da Eva populations are more closely related evolutionarily, which reinforces the possibility that Recife individuals are ancestrally linked to individuals from the northern margin of the Amazon River.

**Fig. 4:**
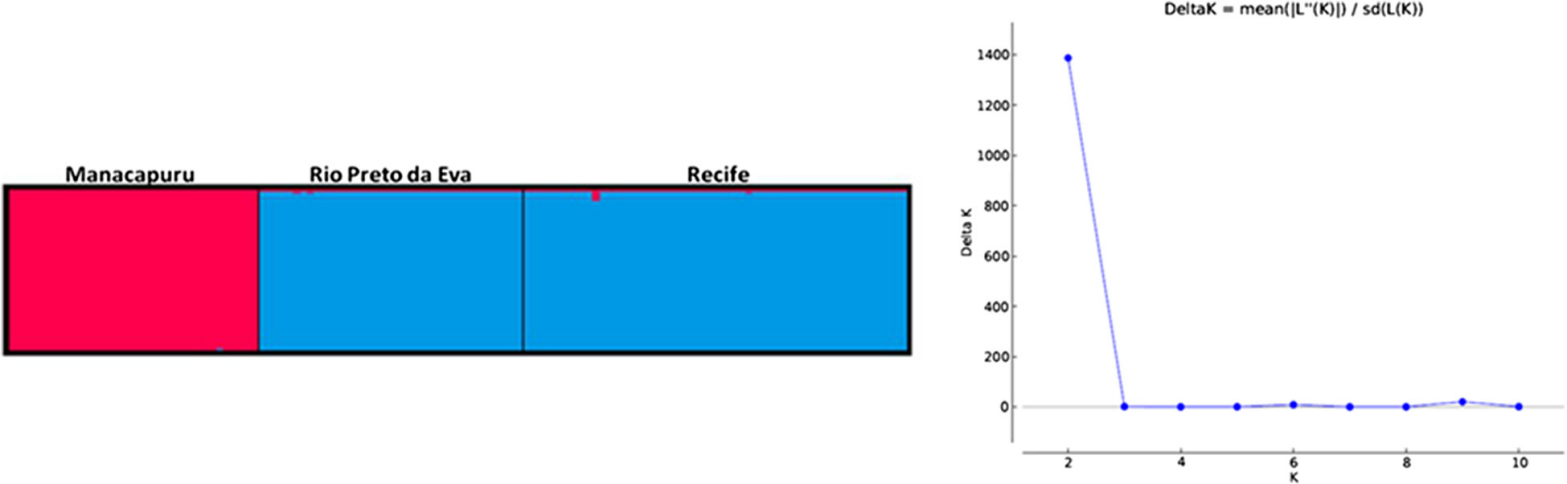
Bar plots and Δ*K* values ranging from 1 to 10 by the STRUCTURE software, inferring the genetic structure of the *L. umbratilis*. The Manacapuru specimens were assigned to the red group, and the Rio Preto da Eva and Recife specimens were assigned to the blue group. The Evanno method predicted that the most likely number of populations was two

Genetic-structure analysis indicated that the populations studied divide into two main subgroups, with the *ad hoc* quantity supporting the number K = 2. Cluster analysis corroborated the separation of the samples into two clades (Fig. 4). Bayesian inference showed that the separation of these two clades occurred between 0.79 and 1.68 Mya—sometime between the Pleistocene and Pliocene.

**Fig. 5:**
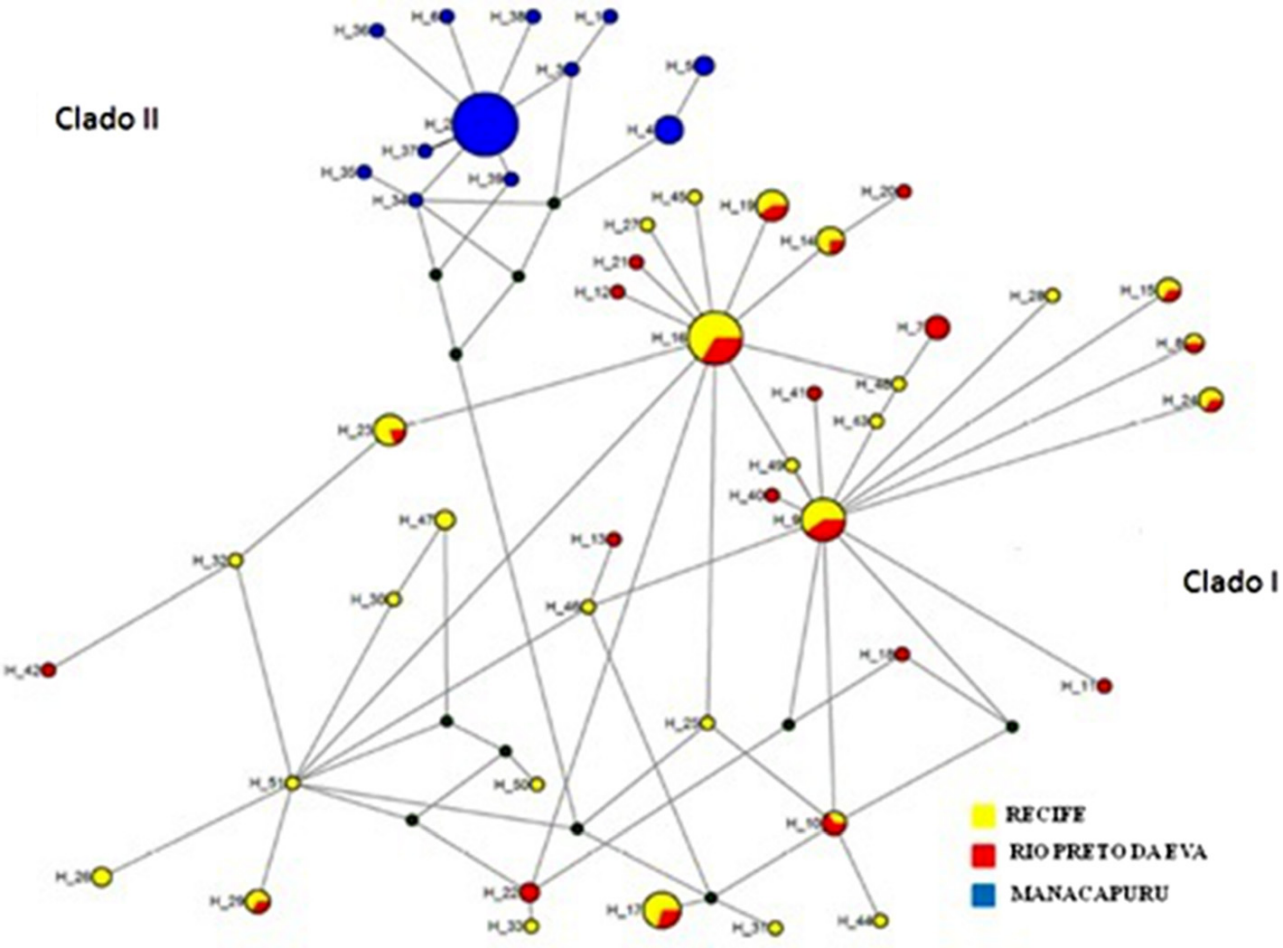
Haplotype network of *L. umbratilis* showing 51 interconnected haplotypes. The size of the circles are proportional to the number of individuals observed for each haplotype. The small circles (green) represent mutational events lost during the evolutionary process

There were 51 alleles observed across the three populations. The most frequent haplotypes were H2, shared by 21 Manacapuru individuals, and H16, shared by 15 Recife and Rio Preto da Eva individuals (Fig. 5). The greatest number of haplotypes was observed in the Recife and Rio Preto da Eva populations representing 76.4 % of the total alleles, which shows that these populations possess a higher level of genetic diversity.

Intrapopulational analysis of *L. umbratilis* specimens revealed a higher level of nucleotide and haplotype diversity in specimens from Rio Preto da Eva and Recife, than in specimens from Manacapuru (Table 1).

**Table 1:** Intra-population genetic diversity measures for each sample

Samples	Ts/Tv	NS	Hd	*Π*
Manacapuru (AM)	10/8	18	0.65556	0.003539
Rio Preto da Eva (AM)	26/1	26	0.95875	0.004870
Recife (PE)	26/2	28	0.94545	0.004807
Total	49/19	66	0.94755	0.019231

*Ts/Tv* transitions/transversions, *NS* Number of polymorphic sites *Hd* Haplotypic diversity, *π* Nucleotide diversity

In addition, the Rio Preto da Eva and Recife populations exhibited a higher level of differentiation than the Manacapuru population, which reflects the high level of genetic divergence that exists between the two clades (Table 2).

**Table 2:** Genetic differentiation among samples

Populations	Kxy	*F*st	Dxy	Da	Ss	Sf
Manacapuru X Rio Preto da Eva	24.41228	0.89677	0.04089	0.03669	2	17
Manacapuru X Recife	24.09141	0.89357	0.04935	0.03618	2	16
Rio Preto da Eva X Recife	2.89617	0.00261	0.00485	0.00001	15	0

*Kxy* Average nucleotide pairwise differences between two groups, *Fst* Pair-wise genetic differentiation, *Dxy* Average number of nucleotide substitutions per site between populations, *Da* Number of net nucleotide substitutions per site between populations, *Ss* Number of shared polymorphisms between pairs of populations, *Sf* Number of fixed differences between pairs of populations

Tajima’s *D* test was negative and significant (*P* <0.05) for all populations, which shows deviation from the neutrality model. This result could be explained by the high number of rare haplotypes in these populations, which would reinforce the possibility of recent expansion or positive selection. Fu’s *Fs* was also negative and significant (*P* < 0.01) for all populations, which confirms the hypothesis of recent expansion (Table 3).

**Table 3:** Neutrality tests and population expansion parameters estimated for each sample

Samples	*Tajima’s D*	*Fu’s Fs*	*R*	SSD
Manacapuru (AM)	−1.70806*	−4.44368**	0.07692	0.02268
Rio Preto da Eva (AM)	−1.81708*	−16.57669**	0.03686	0.00190
Recife (PE)	−1.73173*	−23.45417**	0.02872	0.00054

*R* raggedness index, *SSD* sum of squared deviations

*Tajima’s D* (*p < 0.05); *Fu’s Fs* (**p < 0.001)

The Mismatch distribution also confirms that these populations have recently undergone a process of demographic expansion. The raggedness index (*r*) did not reject the null hypothesis of recent demographic expansion. The sum of square deviation test (*SSD*) was not significant. The haplotype fixation index (Fst) was significant (0.89677 – 0.89357) when comparing the Rio Preto da Eva and Recife populations with the Manacapuru population (Table 2)—this reflects a high level of genetic divergence between these two groups.

### Geometric morphometry

**Fig. 6:**
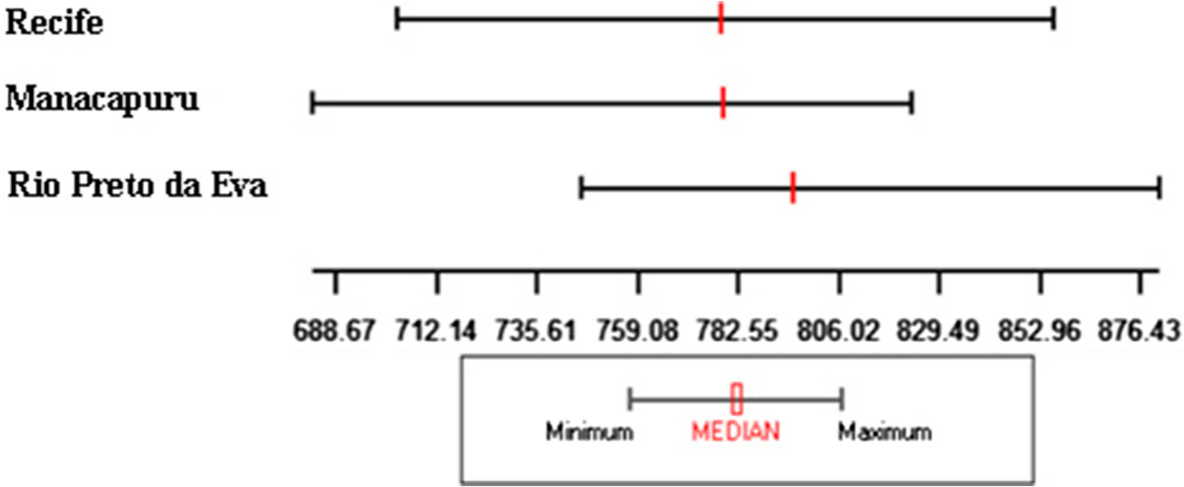
Centroid size variation in the wings of *L. umbratilis* populations from Recife, Manacapuru, and Rio Preto da Eva

Differences in wing size were observed among the three populations studied. The median centroid size of Rio Preto da Eva specimens was larger than specimens from Manacapuru and Recife (Fig. 6). The mean centroid size of Rio Preto da Eva was significantly different from Manacapuru (*p* < 0.05; ANOVA + Tukey’s pairwise comparisons), but not from the Recife population (*p* > 0.05; ANOVA + Tukey’s pairwise comparisons). Also, no significant difference in the mean centroid size was observed between Recife and Manacapuru populations (*p* > 0.05; ANOVA + Tukey’s pairwise comparisons). The allometric effect was 17.18 % (*p* < 0.0001), and it was removed from the shape analysis.

**Fig. 7:**
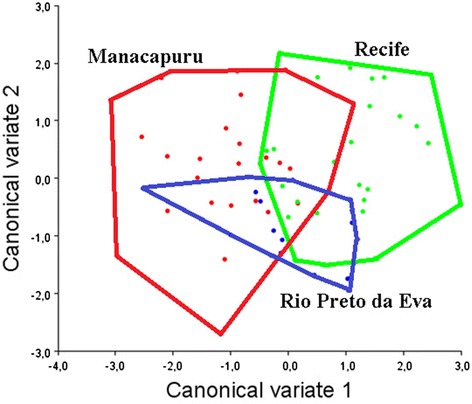
Morphological space of canonical variates derived from comparison among the three *L. umbratilis* populations. Rio Preto da Eva, north of the Amazon River (blue); Manacapuru, southeast of the Negro River (red); and Recife, in the Northeastern Region of Brazil (green). Colored circles represent the individuals from every population studied and polygons represent the clustering of the populations

The canonical variate analysis for the wing shape showed that individuals were clustered into distinct groups in the morphospace according to each population (Fig. 7). Manacapuru and Recife were forming two groups slightly overlapped, whereas Rio Preto da Eva was highly overlapped with the other two populations. The Mahalanobis distance between Manacapuru and Recife populations were 2.0604; and between Manacapuru and Rio Preto da Eva was 1.6548. Rio Preto da Eva and Recife showed the lower distance (1.5246) (*p* < 0.0028 in all comparisons using 10.000 permutations).

## Discussion

Analysis indicated the presence of two distinct clades in *L. umbratilis*: Manacapuru (Clade I), and Rio Preto da Eva and Recife (Clade II). Naturally infected populations of *L. umbratilis* have been identified in Rio Preto da Eva [31]. The fact that the Recife and Rio Preto da Eva populations are so similar, may suggests that *L. guyanensis* could be capable of infecting the Recife population. This highlights the necessity of studies that seek to ascertain the vector capacity of the Recife population of *L. umbratilis*.

The lack of a main natural reservoir (*Choloepus didactylus*) in the Atlantic forest could act as a limiting factor in the biological cycle of *L. guyanensis*. Due to the absence of records of this etiological agent in the state of Pernambuco, *L. umbratilis* has not been incriminated as vectors of ATL despite the high number of cases [3].

The genetic difference observed between Rio Preto da Eva (north of the Amazon River, Amazonas) and Manacapuru (south of the Amazon River) populations in this study could be related to changes in the course of the Amazon River [32]. The analyzes suggest that changes in the course of the Amazon River initially isolated the *L. umbratilis* population from Manacapuru for a long period. According to Haffer [33], separation and a long period of isolation along opposite margins of the river must have had a crucial impact on the speciation of several groups of organisms in the region. This geographic isolation could have caused genetic and bionomic changes in the population from Manacapuru (south of the Amazon River) when compared to the population from Rio Preto da Eva (north of the Amazon River) [14, 15]. This vicariant event happened with other species in this region, such as monkeys and birds [34].

During this long period of separation among *L. umbratilis* populations from the central Amazonian, the populations from Rio Preto da Eva (north of the Amazon River) and Recife (Northeast region) remained possibly linked by Amazon and Atlantic forests until the last glacial period. The presence of a clade formed by Rio Preto da Eva and Recife individuals suggests that these populations diverged more recent, possibly during Pleistocene, as occurred in *Lutzomyia whitmani* [35]. Ready *et al.* [35] assessed *L. whitmani* populations of North and Northeast regions suggesting that there was a continuum of intercrossing between the Amazon and Atlantic forests. This data is supported by our geometric morphometry analysis of wings. These results were unexpected because of the distance, lack of continuity and environmental differences between Amazon and Atlantic forest. The occurrence of several Amazon species of sand flies in Pernambuco reinforce the idea of a continuum between the Amazon and Atlantic forest [36, 37]. However, ecological vicariance should have contributed to the segregation of these populations, primarily via geological and climatic changes—as seen among other organisms in the Amazon region [34].

Fluctuations of climate and vegetation could have modified the distribution of tropical forests during the Cenozoic, resulting in the retraction and expansion of Amazon vegetation [38]. This process could have contributed to the formation of passages between the Atlantic and Amazon forests. This is a possible explanation for the high level of genetic similarity between the North and Northeast populations. The same scenario was observed in the *L. whitmani* and *L. longipalpis* species [39, 40].

In the taxonomic analysis of the *L. umbratilis* complex, phylogenetic analysis showed that the COI marker used had a high discriminatory capacity; thereby demonstrating the presence of two clades within the Central Amazon and Northeast populations. This gene was used previously to study the *L. longipalpis, L. umbratilis*, *Anopheles albitarsis* and *Triatoma brasiliensis* complexes with considerable efficiency [15], [41, 42], [43–45]. The applicability of this mitochondrial marker was bolstered by statistical support from the maximum likelihood tree, which detected the presence of two clades for *L. umbratilis*.

The divergence time estimate indicates that the two clades diverged approximately 0.89 Mya (0.79 – 1.68 Mya), during the Pleistocene. Scarpassa and Alencar [15] obtained similar results when they analyzed the Northern populations. Therefore, more molecular data will be crucial for establishing a more reliable divergence time estimate.

As with molecular data, GM analysis indicated two distinct groups between the studied populations, with some homogeneity among them. Also, the canonical variate analysis of wing shape indicated that Rio Preto da Eva population is significantly closer to Recife population, and otherwise Manacapuru and Recife populations were more distant. These results indicate divergence between *L. umbratilis* inhabiting Manacapuru and Recife/Rio Preto da Eva populations. Similar studies using GM and molecular analysis using *Lutzomyia shannoni* individuals showed divergence between Mexico and USA populations [46, 47].

## Conclusion

The markers used in this study (phenotypic and genotypic) allowed the two clades to be differentiated: Manacapuru (Clade I), and Rio Preto da Eva and Recife (Clade II). The genetic similarities shared by the Recife and Rio Preto da Eva populations suggest that Recife individuals could have a vector capacity similar to individuals found north of the Amazon River. However, we cannot be certain that the Recife population possesses vector competence for the transmission of ATL etiological agents. Our evolutionary analysis highlights the necessity for novel studies and for constant surveillance of the *L. umbratilis* population found in the Atlantic Forest remnant in the state of Pernambuco, Northeastern Brazil.

### Ethical approval

Ethical approval was not required for the current study.
